# Qualitative Pilot Study: Challenges for Primary Healthcare Providers Caring for Refugees in Northeast Ohio

**DOI:** 10.7759/cureus.12572

**Published:** 2021-01-08

**Authors:** Mackenzie J Reece, Sarah Rubin

**Affiliations:** 1 Department of Social Medicine, Ohio University Heritage College of Osteopathic Medicine, Cleveland, USA

**Keywords:** key words: health care delivery, access to care, qualitative research, primary care, immigrant health

## Abstract

Background: Refugees resettling into the United States are faced with complex barriers to accessing basic health care. Qualitative research is needed from the primary health care providers' (PHCP) experience caring for refugees. Examination of PHCPs’ experience adds to a holistic understanding of the healthcare needs of refugees and points to specific health system interventions to improve care. Consideration for Patient-Centered Medical Homes (PMCH) within refugee communities is advanced.

Objective: Gather experiences through narratives from PHCPs to understand challenges and barriers in meeting the health care needs of refugees and suggest solutions.

Design: Qualitative, descriptive framework. Open-ended, semi-structured interviews.

Participants: In-depth interviews (n=seven) with current licensed PHCPs (four physicians and three family nurse practitioners) working in clinic practice settings throughout Northeast Ohio, providing care to four or more refugee families per week.

Approach: Interviews were recorded and transcribed. Transcripts were coded and analyzed utilizing thematic analysis to identify themes.

Key results: Three themes related to challenges faced by PHCPs: 1) coordination and comprehensive care, 2) accessibility of services, 3) provision of patient-centered care.

Conclusions: The challenges PHCPs describe in delivering healthcare to refugee families were physical access to resources and care coordination. Support was found for inclusion of PCMH within refugee communities.

## Introduction

Globally, 71.4 million people are forcibly displaced due to war, violence, poverty, and fear of persecution [1]. From 2000 to 2016, Northeast Ohio settled 7,649 refugees [2]. It is estimated that 500-700 refugees arrive in the Greater Cleveland area annually, predominantly from Bhutan, Ukraine, Iraq, and Somalia [2]. 

Refugees commonly present with a unique constellation of musculoskeletal and pain issues [3-5], infectious diseases [3], common chronic health conditions [4], and mental and social health issues [4-6] related to resettlement and prior traumatic experiences. Barriers in language with low health literacy present further challenges [7]. Additionally, medical staff’s lack of cultural awareness and sensitivity to refugees’ health beliefs and practices undermines care effectiveness [6]. Proximity to health services, securing stable transportation, and navigating the insurance sector present additional obstacles [7].

In contrast to the plethora of evidence of barriers to effective healthcare from the refugee-patient perspective, research aimed at understanding the challenges healthcare providers face caring for refugees has primarily been conducted outside of the United States (US), leaving a problematic gap in our understanding of refugee healthcare in the US [8]. A meta-synthesis of 357 health providers caring for forced and voluntary refugees in the United Kingdom identified challenges in five main areas: strained organizational systems, overburdened resources, unmet cultural sensitivity training, needed professional support, and fragmented care. Mainstreaming refugee health services was suggested to connect regional health specialty services to community care centers [8]. The meta-synthesis highlights important themes, but a more localized approach is needed due to the differences in national and regional healthcare systems. 

The purpose of this qualitative study is to explore US healthcare providers’ challenges in refugee care. Results provide evidence in support of Patient (Refugee) Centered Medical Homes. Comprehensive, patient-centered, coordinated, and accessible care are pillars supported in the Patient-Centered Medical Homes (PCMH) model and in this study [9].

## Materials and methods

The objective of this research is to gather the experiences of primary healthcare providers (PHCP) caring for refugees to better understand their experiences caring for this uniquely vulnerable population. In this paper we present pilot data on PHCPs’ perceived challenges to care delivery and suggestions for improvement; in the discussion, we offer a strategy for improvement of refugee healthcare in the United States that builds on interview data. 

The study design was qualitative in order to understand better the provider experience of refugee care and to interpret the meanings providers give to their beliefs and behaviors [10]. We used in-person interviews to elicit PHCPs’ narratives; our analysis sought emergent themes and was, in this pilot phase, mainly descriptive [11].

Sampling of subjects was purposeful and opportunistic: because of the small total population meeting our inclusion criteria, we contacted local institutions through a local refugee care network and recruited participants through their internal listservs. In addition, we asked potential participants to refer their colleagues to our study, a method referred to as snowball sampling [12]. 

Participants were screened based on inclusion criteria of: 1) currently licensed PHCPs (family nurse practitioners, physician assistants, and physicians) working in Northeast Ohio and 2) providing care directly to four or more refugee patients per week. The research protocol was approved by Ohio University’s IRB protocol #17-X-356.

Participants that met eligibility criteria represented a total of five separate clinics spread throughout Northeast, Ohio. The final sample included four physicians (one osteopathic and three allopathic) and three family nurse practitioners across five family practice clinics (Table 1). Signed consent was taken and all were compensated based on their employers’ regulations with IRB approval. 

**Table 1:** Patient demographics DO: Doctor of Osteopathic Medicine FNP: Family Nurse Practitioner MD: Doctor of Medicine

Participant	Title	Credential	Specialty	Years of experience	Additional Languages spoken
1001	Physician	DO	Family Medicine	9	Yes
1002	Nurse Practitioner	FNP	Family Medicine	7	No
1003	Physician	MD	Family Medicine	6	Yes
1004	Physician	MD	Family Medicine	6	Yes
1005	Nurse Practitioner	FNP	Family Medicine	14	No
1006	Physician	MD	Internal Medicine	16	Yes
1007	Nurse Practitioner	FNP	Family Medicine	4	No

Data collection

Data was collected from March 2018 to May 2018. This study utilized in-person semi-structured, in-depth interviews with pre-screened participants. Pilot interviews were performed by one investigator (M.R.), all ranged from 40 to 60 minutes and followed a list of topics pre-selected by the researcher that focused on providers’ background, challenges, satisfaction, and opinions on healthcare improvements.

Interviews followed a semi-structured design, meaning that while the interviewer presented topics, the participant guided the depth and direction of the conversation; therefore, interviews were participant-centered and emphasized emergent topics meaningful to the participant [13]. Number of interviews was guided by thematic saturation; saturation was reached (no new themes emerging in interviews; no new codes emerging through analysis) after four interviews [11].

Data analysis

All interviews were recorded and transcribed utilizing a professional transcription service (Rev, San Francisco, CA, USA). For the pilot phase, thematic analysis was performed independently by two researchers (first author and research assistant) who then met to clarify discrepancies and create a shared code book. Thematic coding refers to the identification and labeling of interview content systematically with attention paid both to the explicit referent and its context [14]. Our thematic approach was inductive, allowing for emergent themes rather than those imposed by the research question [15]. 

## Results

Participants that met eligibility criteria represented a total of five separate clinics spread throughout Northeast, Ohio. The final sample included four physicians (one osteopathic and three allopathic) and three family nurse practitioners across five family practice clinics (Table 1). The diseases treated included but not limited to: chronic disease management, preventative care, acute care, and infectious diseases.

Three themes relevant to the providers’ experience and challenges faced in refugee health care emerged: 1) coordination and comprehensive care; 2) accessibility of services; and 3) provision of patient-centered care. Our results include quotes from individual participants that represent a theme and though they are not exhaustive, we believe it is incumbent upon the authors to represent the seven interviews through thematic coding with quotes that are best elaborated through the participant's words. 

Coordination and comprehensive care

PHCP cited several challenges in coordinating the healthcare services necessary to provide the comprehensive care their refugee patients needed, including lack of bilingual health education materials and interpretive services to convey important information regarding preventative and diagnostic health care needs. The most frequently cited and seemingly insurmountable problem was the failure of many of their refugee patients to successfully access specialty and follow-up care. Providers understood this problem to be complex and stemming from structural factors. 

For example, few refugees had reliable private transportation. In addition to public transit and taxis, they often utilized government sub-contracted urban transportation services to community clinics; however, this form of service is not available to regional sub-specialty health campuses. A lack of reliable transportation was a common reason the refugee patients reported missed appointments, as PNC providers sympathized, but recognized that a high “no-show” rate became a barrier in itself. 

Providers reported that refugees struggled to navigate the hospital systems where specialist appointments were commonly held, as they were often located in large hospitals with unshared languages: 

“…even if they make it to the building, navigating that gigantic labyrinth of the hospital can be tricky. And if there's not somebody there who is willing to help this person find the room that they're supposed to be in, that can be tricky.” (1002)

To ease problems coordinating care, PNC providers endeavored to schedule specialist appointments for their refugee patients. They recognized that refugee patients missed appointments for many reasons; however, the time to schedule and reschedule appointments based on those reasons was often not available during the office visit.

“We definitely have a hard time with getting patients up to specialists, because they can't really make the appointments for them but it's like a total struggle because there's a million referrals and it takes a while to make them… We make the appointments for them, but then a lot of them don't understand the concept of canceling. They just don't show up.” (1007)

The providers were often left feeling vulnerable and frustrated, as some specialists would not accept referrals from their clinics as a result of the consistent “no-shows”. 

“We have other clinics that are getting irritated because our patients just totally aren't showing up… and then they don't want to see our patients anymore which is total struggle too.” (1007)

When patients did attend referral appointments, providers experienced challenges communicating with specialists due to problems sharing electronic health records (EHRs) between clinics. This placed the burden of communication on refugees. As one provider explained,

“It's really convenient and nice when you have [synchronous EHRs]. It's really hard when they're going to [a hospital without synchronous EHRs]… it's really hard to work with a refugee patient when you don't have access to their reports and then you have to get into faxing permits for them to give you the information back.” (1005)

Providers described a range of problems between refugees and specialists including low-quality translation, improper/unnecessary testing, and a general lack of patience with refugee patients. Trouble receiving adequate specialist care impacted the PHCP’s trust and rapport with their patient and left them feeling unable to provide optimal care:

“[Reference to an experience of improper testing, and recurring appointments] Unresolved, just over and over, and this patient is so frustrated. Anyway, I can't blame the patient for not even wanting to go anymore, because they're not resolving the situation.” (1001)

In sum, PHCPs perceived complex reasons for their refugee patients’ barriers to specialist/follow-up care. Furthermore, they found the problems impossible to address or resolve within the confines of the health encounter. 

Accessibility of services

Providers were challenged by a limited availability of health care supplies and services needed to provide point-of-contact care to their refugee patients, such as pharmacy and mental health services. Providers were also challenged by a limited number of available case managers deemed essential to care:

“It’s not that we don’t help them but it’s difficult and we have to tell them to go see their case manager and if they don’t have one. I mean, this kind of stuff just shouldn’t even exist.” (1006)

Health care resources that were available were often undermined or complicated by refugee patients lacking the insurance and/or funds. PHCP often attempted to mitigate the challenges by offering affordable alternative medical treatment whenever possible. However, without health insurance of any form PHCP felt powerless:

“They don't have much earning…the whole family lives in the same house. And they have to share whatever income they have and if they don't have insurance then I am completely tied up. I can't do anything without their insurance.” (1006)

In addition to the financial burdens placed on refugees and their families, comprehensive care was undermined by lack of insurance: 

“…I can recommend what's recommended, but that wouldn't benefit. Just talking to the patient wouldn't benefit. They need some medications and investigations. Without that, nothing moves forward.” (1006)

Providers found providing necessary educational counseling on needed medical treatments and services challenging due to the limited health literacy of many of their refugee patients. Furthermore, the lack of adequate interpretive services made information exchange in general a continuous challenge.

[Reference to obtaining prescription refills] "Oh no, I'm out. It's done. The medication's done." I'm like, "No, it's not done. And now today, our visit's kind of wasted, because you didn't take your medication." I think medications are a really big challenge for the population in general, but for [providers] too, because I mean, we're responsible for [our patients].” (1007)

PHCPs' were particularly challenged communicating around chronic disease management; they found their refugee patients reluctant to change health practices when they “felt fine” and lacked quality interpreter services to have these more nuanced conversations. 

Providing patient-centered care

PHCPs found it difficult to enact their vision of excellent care, which included establishing trust, robust communication exchange, respecting cultural needs, and managing time. They emphasized the importance of establishing trusting therapeutic relationships to ensure adherence to prescribed plans. Refugee patients presented a unique challenge because of their previous problematic or traumatic experiences in various health systems along their journey. Available time during medical encounters to build therapeutic relationships and trust was limited. 

“The first challenge that we're up against is just kind of building trust. Just kind of establishing that therapeutic relationship. Up until this domestic screening, every health screening that they've done in terms of being a refugee has been exclusionary.” (1002)

Providers felt that factors essential in establishing a successful, trusting relationship included a linguistic and cultural match to facilitate the disclosure of personal information. In addition, they found they were able to care for refugee patients when they clearly explain their role, employed open-ended questions, and showed an interest and basic understanding of their cultural norms.

“You know, you sort of think that they're going to know who you are when you walk in the room, and a lot of them don't…I always introduce myself. I tell them what my role is here. I tell them what we do, what we don't do… I try to be very simplistic and very simple because you can't be too complicated. They'll lose the message.” (1001)

Establishing trust increased engagement between refugees and PHCP during their initial screening encounter. Providers felt validated when refugees and their families returned to receive primary healthcare.

[How do you know if you’ve gained your patient’s trust?]: “Usually not until they come back to see us for primary care. Then when they come back for the established care, primary care visit, a few weeks or a few months later, that's when they've had time to process and might ask questions.” (1002)

Communication was cited as a major challenge, specifically, language mismatch, limited health literacy, and time-consuming dial-in interpretative services. PHCPs understood that translation was not merely a linguistic issue, but context and culture were also critical: 

“I will say having a good translator who is not just a translator but who is truly an interpreter because that’s very different and a translator who translates word for word often is worse. It can be worse than not having a translator.” (1004)

Language and cultural disconnects were seemingly exacerbated during certain types of clinical encounters; for example, was difficult to overcome cultural beliefs and practices that came in conflict with chronic care guidelines:

“The idea of chronic illness is foreign in a lot of cultures…if you're being treated with a medication in the Congo that's for an infection, you take it until the medicine runs out and then you're cured. But if you have hypertension, and I want you to keep filling that bottle up and keep taking the pill, "But I feel fine. I don't need that, I feel fine," that can be hard to communicate.” (1002)

PHCPs found it critical to identify the decision-making authority in refugee families to ensure health directives were followed. But then, when key members were present, acquiring family support taxed an already time-strained health encounter.

“A lot of times I need to involve the family in patient care. Without the family support there's just only so much I can do… if the family is not supporting there's very little that we can do. They need the help of their families. Sometimes the families are not supportive” (1006)

PHCPs were also faced with the challenge of time constraints that were exacerbated by communication and language barriers and high caseloads. As a result, PHCPs felt stressed and hurried through the health encounter. 

“We experience burnout… every five minutes you’re being pulled in another direction. It makes it harder to stop and just approach a patient with a positive attitude and to focus on that patient and just look at them as like a person and try to listen to their ideas about what’s going on and talk with them and explain with them your ideas about what’s going on.” (1006)

Although clinics provided increased time for refugee patients, providers were still challenged to provide optimal care.

## Discussion

Resettled with complex medical and mental health needs, refugees encounter many difficulties navigating the healthcare system. Health care providers likewise face difficulties providing optimal patient-centered care for refugees and their families within the existing healthcare infrastructure. Descriptive interviewing with PHCPs found they were challenged in building trusting relationships, coordinating care needs, securing health resources, and connecting service systems to ensure safe, effective care was consistently provided.

Conceptually, the Refugee-Centered Medical Home (RCMH) provides solutions to barriers found in traditional healthcare systems. Bringing comprehensive healthcare services to refugees and families in their communities affords opportunities to build trust and self-sufficiency by utilizing a collaborative care model. The RCMH constructed within Louisville’s Global Health Program is an example of a refugee care systems model found successful in the provision of comprehensive, accessible, patient-centered care [9]. 

Coordinated and comprehensive care

As PHCPs report, refugees have complex health needs requiring intensive medical services, coordinated across multiple providers and service sectors. Comprehensive coordinated care provided in a collaborative team approach provides a means to close gaps, reduce fragmentation, and address social determinants of health within a community [16]. Health prevention activities to address identified social determinants within a community redirects the operation of a health system from a crisis to a health promotion model. In addition to providing health prevention and wellness promotion, the incorporation of RCMHs provides acute and chronic physical and mental health services to a unique community [9]. 

PHCPs were challenged to bridge gaps in care working in single-purpose primary care centers positioned in proximity to refugee communities. Referrals to outlying care providers and service systems were necessary to meet the complex needs of their patients, yet literacy and transportation barriers and mismatched EHR systems led to high no-show rates often resulting in dismissal and refusal to accept referrals. PHCPs were challenged in communicating the structure and culture of our present health care system. Providers felt refugees were challenged to view disease beyond the presentation of the acute exacerbation; as a result, compliance to the prescribed plan occurred only to the point of symptom abatement without an understanding of disease management. An RCMH would provide the necessary comprehensive care coordination under one roof to blend cultural health beliefs and actualize self-sufficiency.

Accessible services

RCMHs provide accessible services that are convenient for both patients and providers [9]. Providers in this study recognized the challenges of unrealistic time constraints while consistently managing full patient loads. With a lack of patient navigators, providers were negatively impacted in their ability to provide consistent quality care. Providers stated that refugees often have a multitude of unique health conditions requiring additional time for the provider to ensure proper healthcare education and maintenance; however, time was often lost by navigating interpretation services, managing time with full patient loads, and scheduling issues.

Patient-centered care

RCMHs allow providers to build a relationship through shared decision making with the patient and their families’ unique needs, culture, language, values, and preferences [9]. During the refugee patient encounter, PHCPs in the current study were foremost faced with challenges in developing a trusting, therapeutic relationship during the initial screening process and continuation of care due to complex communication barriers and a limited cultural competency. Extensive time was given to ensure appropriate care and education, resulting in going beyond the allotted time for appointments. This increased potential burnout for the physicians as they felt perpetually rushed and behind schedule, as well as vulnerable and frustrated with their perceived limitations in providing excellent care. RCMHs would allot the necessary time and reliable interpreter services contributing to a strong therapeutic relationship. Additionally, RCMHs would facilitate including the patients’ families to enhance patient education. 

Based on our initial results from this pilot study, our future plans are to continue to recruit and interview additional PHCPs in Northeast, Ohio to further strengthen our generalizations. Future studies would examine the specialists’ perspectives and their experiences in the care of refugee patients and the referral process in order to better understand the challenges. 

## Conclusions

Qualitative interviewing revealed systemic structural issues that prevented PHCPs from being able to enact comprehensive, coordinated, patient-centered care for their refugee patients. The results were consistent with a PCMH/RCMH model, which would be especially beneficial to refugee patients in communities where they reside in high concentration.
